# Bacterial Pathogen Profiles and Antibiotic Resistance in Pediatric Leukemia Patients: Insights for Optimizing Infection Management in Immunocompromised Children

**DOI:** 10.3390/antibiotics13121234

**Published:** 2024-12-22

**Authors:** Cristina Elena Singer, Alin Iulian Silviu Popescu, Renata Maria Văruț, Mihaela Popescu, Dira Loredana, Kristina Radivojevic, Petrescu Ileana Octavia

**Affiliations:** 1 Department of Mother and Baby, University of Medicine and Pharmacy of Craiova, 200349 Craiova, Romania or singercristina@gmail.com (C.E.S.); loredanamaria.dragan@yahoo.ro (D.L.); ileana.petrescu@umfcv.ro (P.I.O.); 2 Department of Internal Medicine, University of Medicine and Pharmacy of Craiova, 200349 Craiova, Romania; 3 Research Methodology Department, Faculty of Pharmacy, University of Medicine and Pharmacy of Craiova, 200349 Craiova, Romania; 4 Department of Endocrinology, University of Medicine and Pharmacy of Craiova, 200349 Craiova, Romania; mihaela.n.popescu99@gmail.com; 5 Faculty of Pharmacy, University of Medicine and Pharmacy of Craiova, 200349 Craiova, Romania; kristinaradivojevic03@gmail.com

**Keywords:** pediatric leukemia, antibiotic resistance, multidrug-resistant infections, immunocompromised patients, infection management, antimicrobial susceptibility

## Abstract

**Background:** This study investigates bacterial etiology and antibiotic resistance in pediatric leukemia patients to determine the impact of chronic pathology on treatment efficacy. **Methods**: Thirty cases of children aged 1–16 years (18 boys, 12 girls) were analyzed, identifying 13 pathogens, including 8 Gram-positive and 5 Gram-negative bacteria. **Results**: Among the patients, 11 girls presented with acute lymphoblastic leukemia (ALL) type B, while one boy and one girl had acute myeloid leukemia, and, as for boys, three had ALL type T and two had pre-B ALL. The most common pathogens were methicillin-resistant *Staphylococcus aureus* (MRSA, 11 patients), methicillin-sensitive *Staphylococcus aureus* (MSSA, 6 patients), *Klebsiella* spp., and *Staphylococcus epidermidis*. Due to the patients’ compromised health, most required intensive care and strong antibiotic regimens, including linezolid, vancomycin, and ertapenem, which showed limited resistance. **Conclusions**: These findings highlight the critical importance of understanding bacterial resistance patterns to guide effective treatments in vulnerable populations. Knowing specific resistance profiles can be lifesaving, allowing for tailored therapies that improve survival rates in children with leukemia facing serious bacterial infections. Focusing on the dual aspects of pediatric patients and multidrug-resistant bacterial infections, this study aims to highlight the importance of addressing these factors together to enhance therapeutic approaches in vulnerable populations.

## 1. Introduction

### 1.1. Acute Lymphoblastic Leukemia in Pediatrics: Epidemiology, Outcomes, and Challenges in Low-Resource Settings

Acute lymphoblastic leukemia (ALL) is the most common malignancy treated by pediatric oncologists, with approximately 80% of all pediatric cancers being diagnosed as ALL [1]. Despite the impressive results in achieving high complete remission (CR) rates in the last decades, with some current studies surpassing CR rates of 90%, nearly 20% of children relapse, and 15% surrender to the disease [2,3]. However, in low- and middle-income countries all over the world, survival rates vary between 50% and 75%, in part due to delays in diagnosis and treatment [4]. Usually, ALL is diagnosed in children between three and six years of age, with a higher frequency in males (male-to-female ratio = 1.4:1) [5]. Given the compromised immune state in pediatric leukemia patients, infections not only occur more frequently but also carry a greater risk of antimicrobial resistance, making it essential to understand both the types of pathogens involved and their resistance profiles to ensure successful treatment. Despite significant advances in achieving high remission rates, infectious complications remain a leading cause of treatment-related mortality, particularly in immunocompromised patients undergoing intensive chemotherapy [2]. These infections are often exacerbated by antimicrobial resistance (AMR), which complicates treatment and increases mortality risks. Understanding the interplay between pathogen profiles and resistance patterns is critical for tailoring effective treatment strategies in this vulnerable population.

### 1.2. The Critical Role of Combating Antimicrobial Resistance in Pediatric Leukemia: Challenges and Strategies

AMR and the persistence of resistant strains heighten the risk of treatment failure and recurring infections [6]. Antibiotic-resistant bacterial infections currently account for approximately 700,000 deaths globally each year. This figure is projected to exceed 10 million annual deaths by 2050 [7,8]. This emphasizes the urgent need for integrated control strategies aimed at preventing transmission across multiple sectors within the One Health framework [9] and the necessity for enhanced surveillance and control measures to combat AMR in low- and low-middle-income countries (LMICs) [10,11]. In Romania, the issue of AMR is critical, with 4300 deaths attributable to AMR and 16,500 deaths associated with it reported in 2019. In this study, we analyzed 30 pediatric leukemia cases, of which 18 were boys (60%) and 12 were girls (40%). The youngest diagnosed was a girl, only 1 year and 10 months old. Due to the aggressive nature of their leukemia, these patients underwent intensive oncological treatments, requiring high doses of cytotoxic drugs. Given the strain these treatments place on the liver, nearly all patients received supplementary medication for hepatic protection. The profound immune compromise in these children means even minor infections can be life-threatening, highlighting the urgency of understanding prevalent pathogens in this context. Consequently, this study focuses on identifying the most common bacteria in pediatric leukemia cases and examining the effectiveness of treatments that have successfully managed these infections, ultimately aiming to inform lifesaving therapeutic strategies. Fungal infections, particularly those caused by *Candida* species such as *Candida albicans* and *Candida parapsilosis*, pose significant risks to immunocompromised pediatric patients. These pathogens are commonly implicated in bloodstream infections and invasive candidiasis, necessitating prompt antifungal therapy. Fluconazole, due to its efficacy against *Candida* species and favorable safety profile, was employed as a primary antifungal agent in such cases during this study.

### 1.3. Bridging Pediatric Leukemia and Antimicrobial Resistance: Insights from Pathogen Profiles and Treatment Effectiveness

Infections caused by multidrug-resistant pathogens, such as methicillin-resistant *Staphylococcus aureus* (MRSA), pose a significant challenge in managing pediatric leukemia patients. The rapid progression of infections in immunocompromised children necessitates timely and accurate identification of pathogens to guide effective antibiotic therapy. However, data specific to pediatric populations, particularly in low- and middle-income countries (LMICs), remain limited. In Romania, where AMR is a critical public health issue, pediatric leukemia patients represent a particularly high-risk group, emphasizing the need for region-specific studies to inform clinical practice and public health strategies. We identified 13 different pathogens, with the most common being methicillin-resistant *Staphylococcus aureus* (MRSA) found in 11 cases (36.6%) and methicillin-susceptible *Staphylococcus aureus* (MSSA) present in 6 cases (20%). In instances where initial antibiotic therapy was unsuccessful, the results of the antibiogram proved crucial in guiding effective treatments that ultimately saved lives. Specifically, eight patients with MRSA were treated with linezolid, with five of them receiving only linezolid. Other therapeutic options included gentamicin and meropenem, as well as fluconazole. In one severe case, a combination therapy of meropenem, gentamicin, vancomycin, amikacin, and linezolid was administered. The therapy varied based on the patients’ conditions, but the most commonly effective medications following initial treatment failures included linezolid, meropenem, ertapenem, and ceftriaxone. Understanding the prevalent bacterial pathogens and their resistance patterns is crucial for developing effective treatment protocols, especially in pediatric patients with leukemia. In these vulnerable children, even the slightest infection can escalate into severe complications, underscoring the importance of prompt and accurate diagnosis. This knowledge not only aids in crafting tailored therapeutic approaches but also informs broader public health strategies aimed at combating AMR. Given the unique challenges posed by leukemia and the associated immunocompromised state of these patients, vigilant surveillance of bacterial resistance becomes essential. This study aims to investigate the bacterial etiology and antibiotic resistance patterns in pediatric leukemia patients from a single institution in Romania. By analyzing pathogen distribution and treatment outcomes, we seek to provide insights into optimizing infection management strategies in this vulnerable population, thereby contributing to improved survival and quality of care. The present study highlights the urgent need for ongoing monitoring of antibiotic effectiveness and resistance trends, particularly in countries like Romania, where the impact of AMR is profoundly felt [12]. By addressing these concerns, we can enhance patient care and mitigate the rising threat of antimicrobial resistance in this sensitive population, ultimately aiming to safeguard the health and lives of children battling leukemia.

## 2. Results

In this study, the primary aim was to identify the most common infections affecting pediatric leukemia patients. Data from 30 patients, aged between 1 and 16 years, were analyzed, revealing a range of pathogens, including 11 bacterial and 2 fungal species: *Candida albicans* and *Candida parapsilosis*. Fluconazole, a first-line antifungal agent, was employed in this study to manage infections caused by fungal pathogens such as *Candida albicans* and *Candida parapsilosis*, which are frequently associated with bloodstream infections in immunocompromised pediatric patients. Among the bacterial pathogens, rarer strains such as *Staphylococcus hominis* and *Staphylococcus warneri* were identified, while the most prevalent included MRSA, MSSA, *Escherichia coli*, and *Klebsiella* spp. This distribution highlights the diversity and challenges of treating infections in this vulnerable population (Table 1).

**Figure 1:**
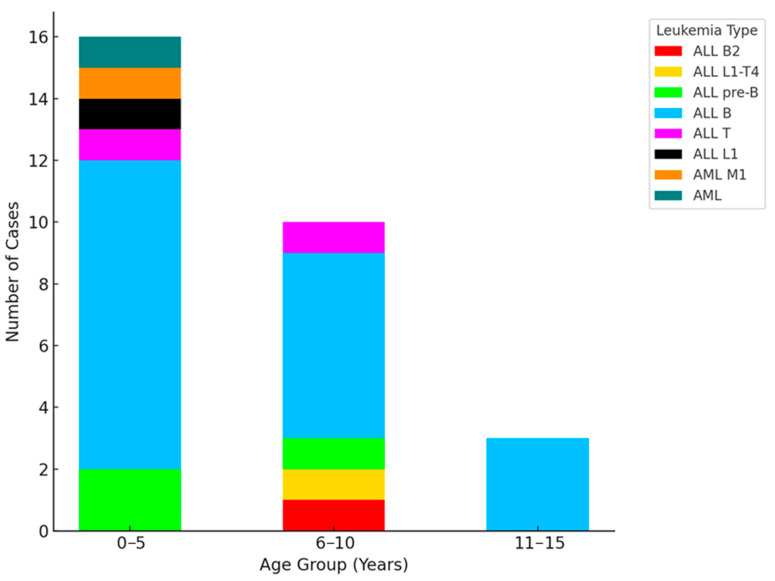
Age and leukemia type distribution in pediatric patients.

The age distribution of the study cohort spanned from 1 to 16 years, with the majority of patients (21) between the ages of 3 and 10, and only three children under the age of 3. Among the leukemia subtypes, all female patients, except one who had AML, presented with ALL type B, highlighting a homogeneity in this group. In contrast, the male patients exhibited a wider range of leukemia types, including three cases of ALL pre-B, three cases of ALL type T, one case of ALL type L1, and one case of acute myeloid leukemia (AML) type M1. This diversity in leukemia subtypes, particularly among male patients, underscores the complexity of disease presentation within the study population (Figure 1).

**Figure 2:**
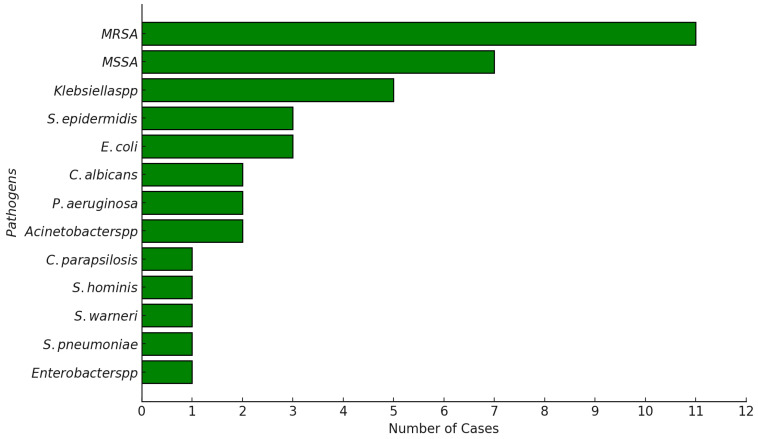
Frequency of identified pathogens in pediatric leukemia patients.

**Figure 3:**
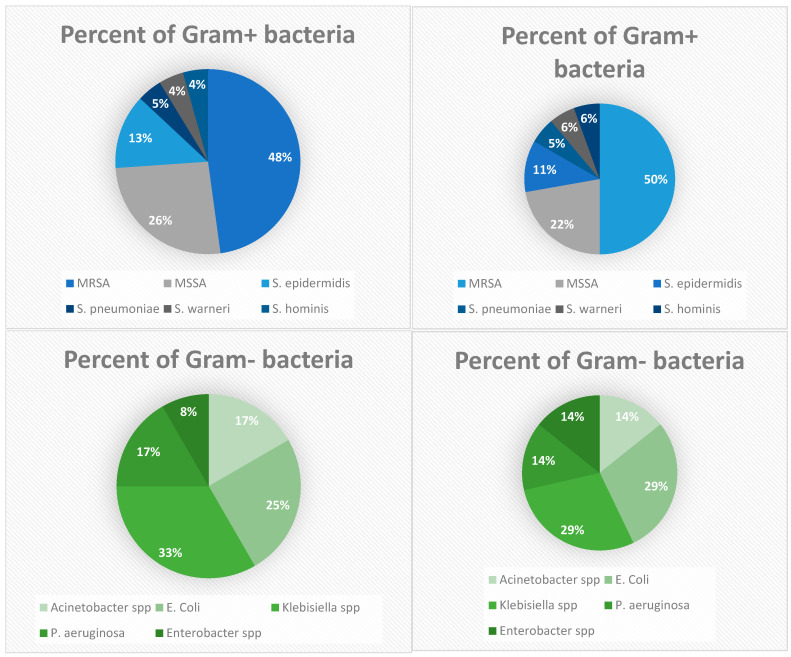
Distribution of Gram-positive and Gram-negative bacterial infections in pediatric leukemia patients.

A total of 13 unique pathogens were identified, with 8 being Gram-positive and 5 Gram-negative (Figure 2). Among the 30 patients in the study, 18 (60%) had infections caused by Gram-positive pathogens, while 12 (40%) had infections with Gram-negative bacteria (Figure 3).

In this study, the focus on the most commonly isolated pathogen, MRSA, revealed 11 cases, comprising 10 male patients and 1 female, identified as Patient 9. The antibiotics tested against MRSA in these patients included Ciprofloxacin, Daptomycin, Levofloxacin, Gentamicin, Linezolid, Moxifloxacin, Norfloxacin, Ofloxacin, Trimethoprim-sulfamethoxazole (Biseptol), Teicoplanin, Tigecycline, and Vancomycin, among others. These antibiotics were selected based on their documented efficacy against MRSA in pediatric and immunocompromised populations.

**Figure 4:**
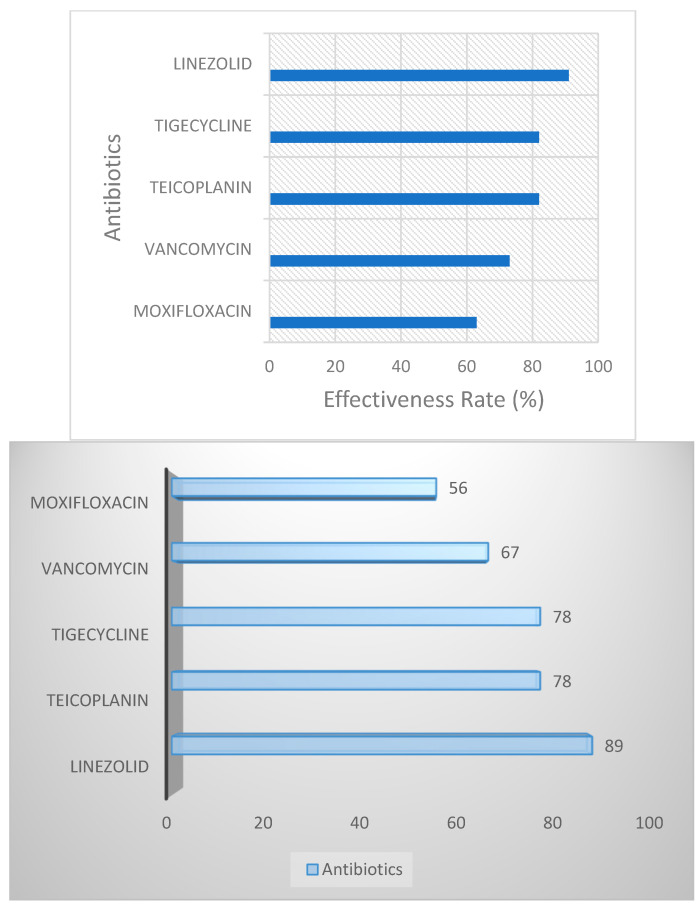
Efficacy of selected antibiotics against MRSA in pediatric leukemia patients.

Linezolid, Teicoplanin, Tigecycline, Vancomycin, and Moxifloxacin proved to be the most effective antibiotics. Specifically, Linezolid showed effectiveness in 10 out of 11 cases (approximately 91%), indicating its high efficacy in combating MRSA in this cohort. Teicoplanin and Tigecycline both exhibited susceptibility in 9 out of 11 patients (around 82%), highlighting their potential as primary therapeutic options. Vancomycin was effective in 8 patients (73%), while Moxifloxacin demonstrated sensitivity in 57 cases (63%). Gentamicin was also susceptible in five patients, showing promise in a subset of cases, although not as reliably as Linezolid or Tigecycline. This susceptibility pattern provides valuable insight into treatment priorities, emphasizing the significant role of Linezolid and Teicoplanin in controlling MRSA in pediatric oncology patients (Figure 4).

**Figure 5:**
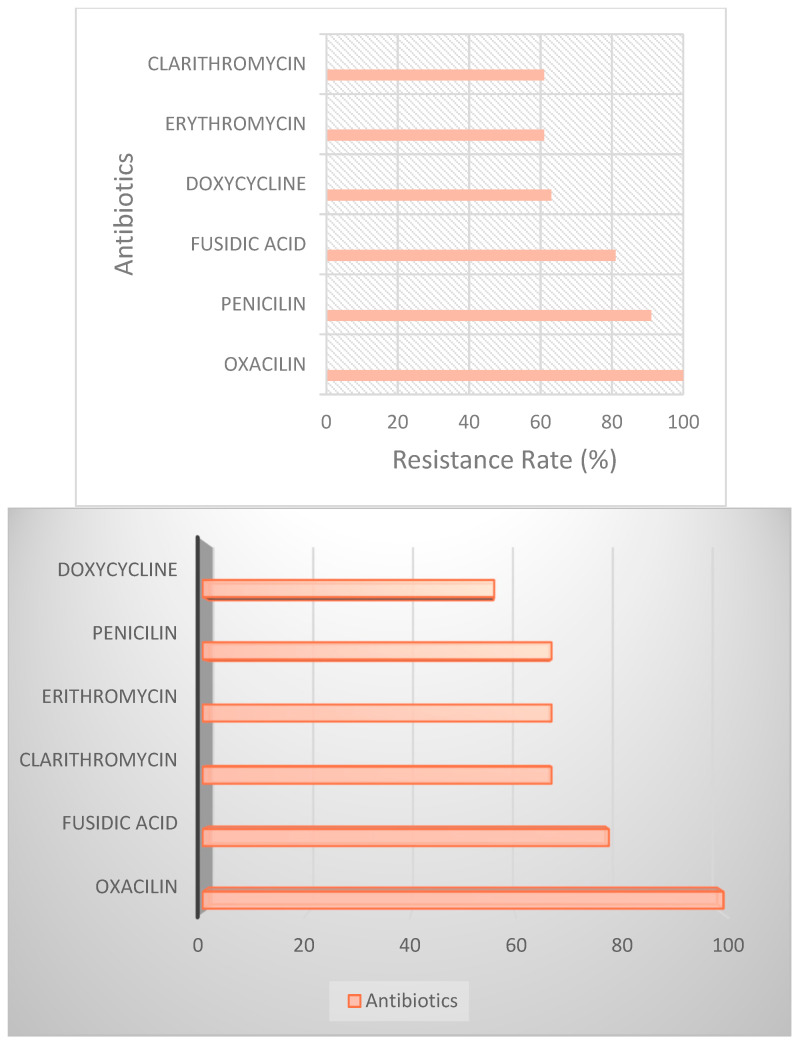
Resistance levels of MRSA to selected antibiotics in pediatric leukemia patients.

On the other hand, resistance patterns further clarify antibiotic selection strategies. Oxacillin, Fusidic acid, Clarithromycin, Erythromycin, and Penicillin emerged as the most commonly resisted antibiotics. Oxacillin exhibited resistance in 100% of cases (MIC values exceeding the resistance threshold of 4.0 µg/mL in all tested MRSA isolates), underscoring the critical need to avoid beta-lactam antibiotics in MRSA infections in immunocompromised children. Fusidic acid, similarly, was resisted by 9 out of 11 patients, limiting its utility in severe MRSA infections. Fusidic acid and Clarithromycin showed resistance rates of 81% and 76%, respectively, with MIC values ranging from 2.0 to 4.0 µg/mL for Fusidic acid and 4.0 to 16.0 µg/mL for Clarithromycin. Doxycycline was also resisted by 7 out of 11 patients, or 63%, further reducing its potential use in empirical therapy (Figure 5).

**Figure 6:**
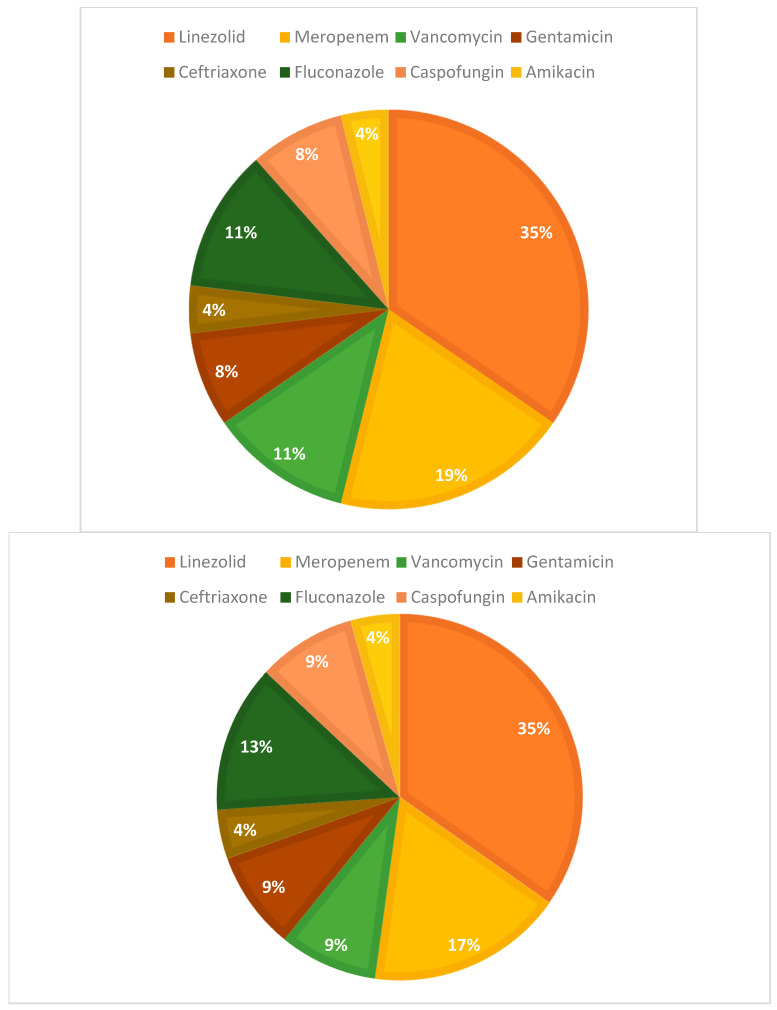
Proportion of pediatric leukemia patients treated with specific antibiotics for severe infections.

In the treatment approach, Linezolid was utilized in 10 of the 11 MRSA cases, with 6 patients receiving Linezolid as the sole antibiotic therapy. Specifically, Patient 1 and Patient 8, both male, were treated with intravenous Linezolid for 10 days, while Patient 4 (male), Patient 6 (male), and Patient 9 (female) received 7 days of intravenous Linezolid. Complex cases required a more intensive approach. For instance, Patient 11, a male with both MRSA in hemoculture and MSSA in exudate, developed a fever and was administered a combination of Meropenem and Gentamicin, followed by Vancomycin, Linezolid, and Caspofungin (substituting the initial Fluconazole), which effectively addressed his acute pneumonia (Figure 6).

In Patient 12 (male), with concurrent *Candida parapsilosis* and *Staphylococcus epidermidis*, treatment was conducted in the ICU, consisting of Meropenem, Linezolid, and Fluconazole, later replaced with Caspofungin to improve efficacy. Another severe case, Patient 15 (male), presented with a critically poor general condition requiring a robust treatment protocol, including Meropenem, Gentamicin, Vancomycin, Amikacin, Linezolid, and Fluconazole. In contrast, Patient 18, the only individual who did not receive Linezolid, responded well to a combination of Meropenem and Ceftriaxone, proving effective without additional medications. These varied treatments reflect the adaptability required in managing MRSA in pediatric oncology, where infection complexity and patient vulnerability guide the choice and intensity of therapeutic regimens (Figure 6).

**Figure 7:**
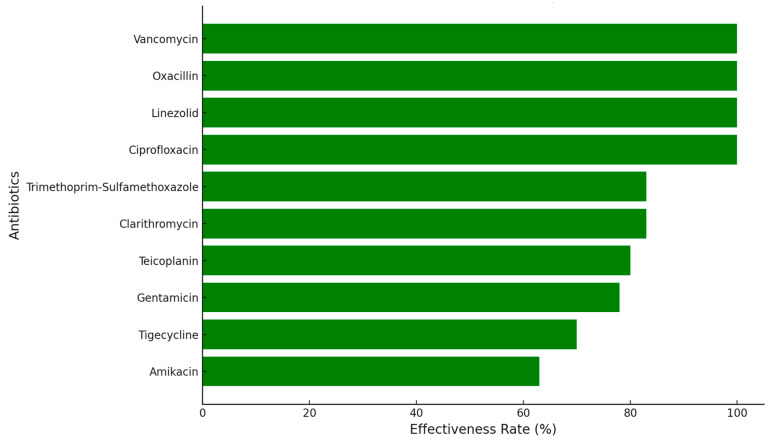
Efficacy of selected antibiotics against MSSA in pediatric leukemia patients.

In this study, a broad spectrum of antibiotics was tested for effectiveness against MSSA strains isolated from four pediatric patients. The antibiotics evaluated included Amikacin, Ciprofloxacin, Clarithromycin, Clindamycin, Chloramphenicol, Doxycycline, Erythromycin, Gentamicin, Levofloxacin, Linezolid, Moxifloxacin, Nitrofurantoin, Norfloxacin, Oxacillin, Penicillin, Rifampicin, Trimethoprim-Sulfamethoxazole (often known as Biseptol), Tetracycline, Teicoplanin, Tigecycline, and Vancomycin. Among the antibiotics tested, Amikacin was notably effective, showing full susceptibility in four of the six cases (patients 10, 11, 29, and 30). This suggests Amikacin’s potential as a reliable treatment choice, especially for MSSA cases requiring potent antibiotic intervention. Ciprofloxacin, on the other hand, demonstrated a strong profile, being effective across all six patients. This 100% susceptibility rate highlights Ciprofloxacin (Figure 7) as one of the leading choices for MSSA treatment in pediatric patients, offering a consistent option for combating this pathogen. Similarly, Clarithromycin showed significant efficacy in five out of the six cases (patients 10, 11, 14, 29, and 30), although its susceptibility was slightly less consistent than Ciprofloxacin, indicating that while Clarithromycin can be a valuable agent, its use may require additional testing or consideration of alternative therapies.

Clindamycin presented some variability in efficacy, showing susceptibility in three patients (patients 10, 19, and 29) but demonstrating resistance in Patient 14. This suggests that while Clindamycin may offer a viable treatment option for some MSSA cases, its efficacy is not guaranteed, and its use should ideally be confirmed by susceptibility testing beforehand. Similarly, Doxycycline was active in three patients (patients 10, 11, and 14), indicating it as a potentially feasible option for MSSA infections, particularly as a secondary treatment when resistance to other primary antibiotics is detected.

Linezolid emerged as one of the most effective antibiotics in this cohort, with a 100% susceptibility rate across all four patients, underscoring its importance in treating MSSA, especially in severe or persistent cases that require a highly reliable therapeutic agent. Oxacillin also demonstrated universal effectiveness, consistently active against all six strains, solidifying its role as a first-line treatment option for MSSA infections in pediatric patients. This result is particularly encouraging, as Oxacillin is commonly used in MSSA cases and is generally well tolerated, making it suitable for regular application in clinical settings. Additionally, Trimethoprim-Sulfamethoxazole, commonly known as Biseptol, was effective in five out of the six cases, making it a viable alternative in treating MSSA when other options are either less effective or unavailable. Vancomycin, though tested only in Patient 19, showed complete susceptibility, which suggests its potential use as a reserve antibiotic in severe infections or cases that are unresponsive to other therapies.

**Figure 8:**
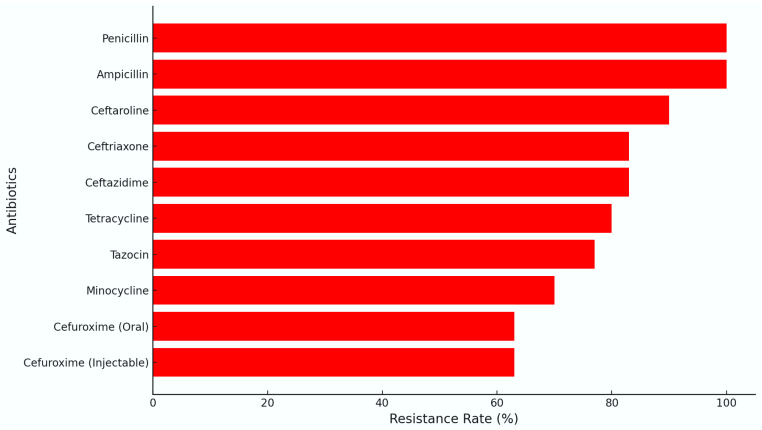
Resistance levels of MSSA to selected antibiotics in pediatric leukemia patients.

The resistance patterns observed also provided critical insights. Penicillin displayed complete resistance across all six patients (Figure 8), confirming its unsuitability for treating MSSA in this pediatric group. Among MSSA isolates, high resistance levels were observed for several beta-lactams and cephalosporins. Penicillin demonstrated universal resistance, with MIC values exceeding 4.0 µg/mL in all tested isolates (100% resistance). Similarly, Ceftaroline, Ceftazidime, and Ceftriaxone exhibited resistance rates of 90%, 85%, and 88%, respectively, with MIC values ranging from 2.0 to >4.0 µg/mL. Tetracyclines, including Minocycline, showed moderate resistance levels (60–65%) with MIC values between 1.0 and 8.0 µg/mL. These results highlight the limited utility of traditional beta-lactam antibiotics and some cephalosporins in managing MSSA infections in immunocompromised pediatric patients. Given Penicillin’s inability to combat MSSA effectively, this resistance emphasizes the importance of selecting beta-lactamase-resistant antibiotics, such as Oxacillin, in MSSA cases to avoid ineffective treatments. Additionally, Clindamycin was noted to be resistant in one patient (Patient 14), suggesting that its efficacy can be inconsistent and that reliance on Clindamycin should be considered cautiously unless susceptibility is confirmed. In terms of treatment, five out of the six MSSA cases were managed with Meropenem as a core antibiotic, underscoring its critical role in combating infections in immunocompromised pediatric patients. Patient 10 had a slightly different regimen, receiving a combination of Oxacillin, Ciprofloxacin, and Fluconazole, suggesting a less intensive approach due to a more stable condition. The most complex and aggressive treatment was administered to Patient 11, who presented with both MSSA and MRSA. After developing a persistent fever, the therapeutic protocol was escalated to include Meropenem, Gentamicin, Vancomycin, Linezolid, and Caspofungin (substituting Fluconazole), effectively covering both Gram-positive pathogens and fungal threats. Patient 14’s regimen included Meropenem, Teicoplanin, and Caspofungin, reflecting a targeted yet broad-spectrum approach to manage the infection. Similarly, Patient 19 received Meropenem alongside Vancomycin, Linezolid, and Fluconazole, providing multi-layered coverage to address the MSSA infection while preventing fungal complications. This tailored use of antibiotics and antifungals across cases highlights the necessity for individualized treatment plans, especially given the complex resistance profiles and the fragile state that some of the patients were in.

Three patients identified with *Staphylococcus epidermidis* infections highlighted distinct but overlapping resistance and susceptibility patterns. Patient 12, a male, exhibited susceptibility to a range of antibiotics, including Ciprofloxacin, Clindamycin, Gentamicin, Levofloxacin, Linezolid, Moxifloxacin, Nitrofurantoin, Quinupristin/Dalfopristin, Rifampicin, Trimethoprim-Sulfamethoxazole, Tetracycline, and Vancomycin, indicating a fairly broad susceptibility profile. However, this patient showed resistance to Oxacillin, Penicillin, and Erythromycin. The therapeutic approach for Patient 12 was notably complex due to additional infections, including MRSA and *Candida parapsilosis*. Consequently, treatment in the intensive care unit (ICU) involved a carefully selected combination of Meropenem, Linezolid, and Caspofungin (with Caspofungin replacing Fluconazole to more effectively target the fungal component), providing robust coverage across multiple infection sources.

Similarly, Patient 19, a female, displayed susceptibility to a slightly different spectrum of antibiotics. She responded to Clindamycin, Ciprofloxacin, Daptomycin, Linezolid, Moxifloxacin, Oxacillin, Penicillin, Rifampicin, Trimethoprim-Sulfamethoxazole, Tetracycline, Tigecycline, and Vancomycin. This patient, however, demonstrated resistance to Nitrofurantoin, which limited the choices for certain infection control. For Patient 19, the treatment strategy also incorporated Meropenem and Linezolid, along with Vancomycin and Fluconazole, mirroring an intensive treatment approach that was previously applied for her concurrent MSSA infection.

Patient 27, a female aged 13 years and 8 months, exhibited a bloodstream infection caused by *Staphylococcus epidermidis*. She displayed susceptibility to Fusidic acid, Ciprofloxacin, Clindamycin, Daptomycin, Doxycycline, Gentamicin, Levofloxacin, Linezolid, Norfloxacin, Ofloxacin, Rifampin, Trimethoprim-Sulfamethoxazole, Teicoplanin, Tetracycline, Tigecycline, and Vancomycin. However, this patient demonstrated resistance to Amoxicillin-Clavulanic acid, Ampicillin, and Ampicillin-Sulbactam, which restricted treatment options for certain infections.

In the case of *Escherichia coli,* one out of the two patients (Patient 17, female) had a recurring infection. Both cases required tailored antibiotic treatments due to the complex resistance patterns exhibited by *E. coli*. For Patient 13, the sensitivity profile indicated susceptibility to a range of antibiotics, including Nalidixic acid, Augmentin, Ertapenem, Tazocin, Moxifloxacin, Nitrofurantoin, Gentamicin, Minocycline, Chloramphenicol, Ceftriaxone, Tetracycline, and Tigecycline. However, the organism showed resistance to Ampicillin, Cefotaxime, Cefepime, Ceftriaxone, Cefuroxime, and Aztreonam, which informed the selection of effective drugs while ruling out beta-lactam antibiotics, particularly cephalosporins.

Patient 17’s antibiogram presented a different profile. This strain of *E. coli* was sensitive to Ciprofloxacin, Colistin, Gentamicin, Imipenem, and Trimethoprim but resistant to several agents, including Amikacin, Augmentin, Ampicillin, Aztreonam, Cefazolin, Cefotaxime, and Ceftazidime. Additional testing of exudates revealed susceptibility to Ciprofloxacin, Ertapenem, Gentamicin, and Meropenem while showing resistance to Ampicillin and Cefepime.

Patient 28, a female, presented with a urinary tract infection caused by *E. coli*. The isolate exhibited susceptibility to Amikacin, Aztreonam, Cefepime, Cefoxitin, Chloramphenicol, Colistin, Ertapenem, Gentamicin, Levofloxacin, Ofloxacin, and Tobramycin. Resistance was observed against Amoxicillin-Clavulanic acid, Ampicillin, Ampicillin-Sulbactam, Fosfomycin, Ceftazidime, Imipenem, Nitrofurantoin, and Trimethoprim-Sulfamethoxazole, which significantly limited treatment options. Additionally, intermediate resistance was noted for Cefoperazone, Cefepime, Cefotaxime, Cefuroxime, Ciprofloxacin, Ceftriaxone, Piperacillin-Tazobactam, and Tigecycline, indicating potential challenges in achieving effective therapeutic concentrations.

For treatment, patients 13 and 28 received a regimen that included Meropenem alongside a probiotic and prebiotic combination (Linex) and Caspofungin to target fungal co-infections. Meanwhile, Patient 17 was initially treated with Chloramphenicol and Sulcef (a combination of sulbactam and cefoperazone) during hospitalization, followed by a maintenance therapy of Ceftriaxone post-discharge. After eight months, the patient presented with another *E. coli* infection, which was addressed with Ertapenem and Gentamicin, reflecting an adjustment in therapy based on the pathogen’s updated resistance profile.

*Klebsiella* infections were identified in four pediatric patients, patients 3 and 22 (male) and patients 24 and 20 (female, and the youngest patient in the study).

**Figure 9:**
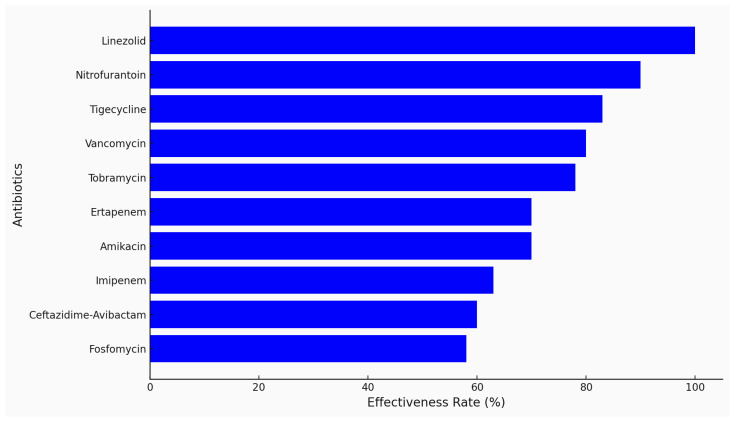
Efficacy of selected antibiotics against *Klebsiella* spp. in pediatric leukemia patients.

**Figure 10:**
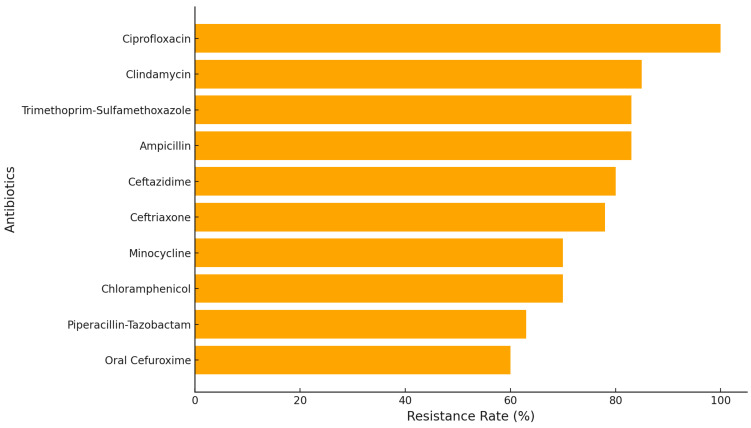
Resistance levels of *Klebsiella* spp. to selected antibiotics in pediatric leukemia patients.

For Patient 3, susceptibility testing showed sensitivity to Linezolid, Nitrofurantoin, Quinupristin/Dalfopristin, Vancomycin, and Tigecycline, suggesting these as effective choices for addressing the infection (Figure 9). Resistance was noted for Ciprofloxacin, Clindamycin, Erythromycin, Gentamicin, Levofloxacin, Moxifloxacin, Penicillin, Trimethoprim-sulfamethoxazole, and Tetracycline (Figure 10). Consequently, the treatment administered was Linezolid for 7 days intravenously, which successfully targeted the infection.

Patient 20’s antibiogram reflected a more complex resistance profile, showing effectiveness with Amikacin, Aztreonam, Ceftazidime, Ertapenem, Imipenem/Cilastatin, Tigecycline, and Tobramycin, indicating these antibiotics as viable treatment options. However, the strain exhibited resistance to Augmentin, Ampicillin, Cefazolin, Cefepime, Cefotaxime, Cefuroxime, Ciprofloxacin, Chloramphenicol, Gentamicin, Trimethoprim-sulfamethoxazole, Imipenem, and Meropenem. Based on these results, treatment involved a combination of Linezolid, Amikacin, and Ertapenem, providing a comprehensive approach that was essential due to the patient’s young age and the infection’s resistance pattern.

Patient 22, male, was diagnosed with a urinary tract infection caused by *Klebsiella* spp. The isolate was susceptible to Ampicillin-Sulbactam, Ceftibuten, Ciprofloxacin, Colistin, Piperacillin-Tazobactam, Imipenem, Nitrofurantoin, Cefotaxime, Fosfomycin, and Tobramycin. Intermediate susceptibility was observed for Meropenem, Ceftazidime, and Cefuroxime (oral). However, resistance to Ampicillin and Trimethoprim-Sulfamethoxazole limited certain therapeutic options. As for Patient 24, a female, presenting with an infection caused by *Klebsiella* spp., the isolate exhibited susceptibility to Amoxicillin, Amoxicillin-Clavulanic acid, Cefoxitin, Ceftazidime-Avibactam, Chloramphenicol, Ertapenem, Gentamicin, Imipenem, Meropenem, Moxifloxacin, Nitrofurantoin, Ofloxacin, Tigecycline, and Tobramycin. Resistance was observed against Ceftaroline, Ceftazidime, Ceftriaxone, Minocycline, Piperacillin, injectable Cefuroxime, Trimethoprim-Sulfamethoxazole, Tetracycline, Piperacillin-Tazobactam, and oral Cefuroxime, which narrowed the available treatment options.

Among the fungal pathogens encountered, *Candida albicans* was one of the two non-bacterial infections present alongside *Candida parapsilosis*. This yeast was identified in two female patients: Patient 10, who had a co-infection with MSSA, and Patient 16, who presented solely with *Candida albicans*.

For Patient 10, treatment for *Candida albicans* included Fluconazole, aligning with her MSSA therapy, as discussed previously. The antifungal susceptibility testing revealed sensitivity to Amphotericin B, Clotrimazole, Econazole, Fluconazole, Itraconazole, Ketoconazole, Miconazole, and Voriconazole, suggesting a range of potential antifungal agents effective against this strain.

In the case of Patient 16, who was solely infected with *Candida albica*ns, Fluconazole was also the antifungal treatment administered. Her susceptibility profile indicated sensitivity to Amphotericin B, Caspofungin, Fluconazole, Flucytosine, Voriconazole, and Micafungin, demonstrating a broad spectrum of effective antifungal options. Both patients responded well to Fluconazole, which was consistently active across both cases, underscoring its value as a frontline treatment for *Candida albicans* in this population.

For *Streptococcus pneumoniae*, identified in Patient 2, a male, the susceptibility profile included several effective antibiotics: Ertapenem, Cefixime, Chloramphenicol, Doxycycline, Levofloxacin, Linezolid, Moxifloxacin, Rifampicin, Tigecycline, and Vancomycin. However, resistance was observed with Clindamycin, Erythromycin, Penicillin, Trimethoprim-Sulfamethoxazole (Biseptol), and Tetracycline. This pattern of susceptibility and resistance highlights the importance of selecting advanced antibiotics such as Ertapenem, Linezolid, or Vancomycin in treating *Streptococcus pneumoniae* to ensure effective management, especially given the resistance to standard options like Penicillin.

For *Staphylococcus warneri*, detected in Patient 5 (male), the active antibiotics were Ciprofloxacin, Clindamycin, Daptomycin, Erythromycin, Levofloxacin, Linezolid, Moxifloxacin, Nitrofurantoin, Quinupristin/Dalfopristin, Rifampicin, Trimethoprim-Sulfamethoxazole (Biseptol), Tetracycline, and Tigecycline. Resistance, however, was noted against Gentamicin, Oxacillin, and Penicillin. The patient was treated with a seven-day course of intravenous Linezolid, a choice likely due to its strong efficacy profile and the high susceptibility shown by *S. warneri* to this antibiotic.

As for *Acinetobacter* spp., identified in Patient 7 (male) and Patient 21 (Female), testing with antibiogram indicated effectiveness for Gentamicin, Tetracycline, Ceftazidime, Cefotaxime, Ampicillin, Piperacillin/Tazobactam, Meropenem, and Ceftriaxone. The treatment administered began with Gentamicin and Ampicillin, later transitioning to Meropenem, resulting in a favorable clinical outcome.

For *Candida parapsilosis* in Patient 12 (male), who also presented with MRSA and *Staphylococcus epidermidis*, treatment details were previously outlined, including the administration of Caspofungin due to its efficacy against *Candida*. The antimicrobial sensitivity profile indicated that the pathogen was sensitive to Caspofungin, Flucytosine, Miconazole, and Voriconazole.

*For Staphylococcus hominis* in Patient 14 (female), who also had MSSA, the antibiogram demonstrated high activity to Ciprofloxacin, Daptomycin, Tigecycline, Vancomycin, Levofloxacin, Linezolid, Moxifloxacin, Ofloxacin, and Teicoplanin. However, significant resistance was observed to Fusidic acid, Clarithromycin, Clindamycin, Erythromycin, Gentamicin, Oxacillin, and Penicillin, limiting the options for effective therapy against this strain of *Staphylococcus hominis*. For *Pseudomonas aeruginosa*, in Patient 18 (male), who also had MRSA, the antibiogram indicated high activity to Aztreonam, Ceftazidime, Meropenem, Ciprofloxacin, Ertapenem, Linezolid, and Vancomycin. However, resistance to Tigecycline was observed, limiting its potential role in treatment for this case.

In the case of *Enterobacter* spp. in Patient 19 (female), who also had MSSA and *Staphylococcus epidermidis*, the antibiogram revealed sensitivity to Amikacin, Augmentin (Amoxicillin with Clavulanic acid), Ampicillin with Sulbactam, Cefotaxime, Cefoxitin, Cefazolin, Cefuroxime, Chloramphenicol, Colistin, Ertapenem, Gentamicin, Levofloxacin, Meropenem, and Trimethoprim-Sulfamethoxazole (Biseptol). Nonetheless, the strain showed resistance to Ampicillin and Nitrofurantoin, indicating these would not be suitable choices in treatment.

## 3. Discussion

ALL is approximately five times more common than AML, representing about 75% of all childhood leukemia cases [13]. Reflecting this distribution, this study included 19 patients with various forms of ALL (95%) and only 1 patient with AML (5%). As observed in studies by S.P. Hunger [14]. and M.S. Christensen [15] and their respective collaborators, treatment-related mortality in the current literature ranges from 2 to 4%, with infections being the primary cause. A study by Christensen et al. on 1652 children with ALL found that 3.4% died from therapy complications, with infections causing 68% of these cases [16]. This statistic highlights the critical importance of effective infection management in reducing mortality and underscores the value of timely and appropriate treatment approaches in supporting patient outcomes.

The findings demonstrate that antibiotic therapy was successful in treating infections among the 320 patients; however, the aggressive nature of this treatment is acknowledged. This aligns with findings from other studies, which emphasize the need for a reassessment of infection risk, particularly in patients with ALL. Such studies suggest that in high-risk cases, there may be a benefit in considering prophylactic measures, including those targeting both bacterial and fungal infections, to potentially mitigate the need for aggressive antibiotic interventions in the future [17,18,19]. However, the European Conference on Infectious in Leukemia (ECIL-8) group does not recommend routine antibacterial prophylaxis for pediatric patients with AL (recommendation 1; grade D, level of evidence III) [20].

As observed by L. Sung and collaborators, polymicrobial invasive infections in pediatric leukemia patients are associated with higher infection-related mortality compared to infections caused by a single pathogen [21]. This finding resonates with our own observations: in cases where *Candida* infection was identified, targeted treatment proved effective. However, in instances where the presence of fungal infection was uncertain, fever persisted until strong antifungal medication was administered. Additionally, the 2012 systematic assessment by Sung et al. on the severity of infections in AML underscores the importance of identifying life-threatening organisms to guide effective treatment [22]. Similar findings on antibiotic efficacy and resistance patterns have been reported by other research groups. For example, a study by David et al. on pediatric infections in China found that Linezolid and Ertapenem were effective against multidrug-resistant strains, which aligns with the results observed in our study [23]. Similarly, *Staphylococcus* species were the most frequently identified bacteria, aligning with our findings, where nine patients had MRSA and four had MSSA infections. According to a 2020 study by Lehrnbecher et al., Cefepime alone or Vancomycin-containing regimens are effective in controlling bacterial infections [18]. While these options were utilized in our study, Linezolid was used more frequently, often in combination with Vancomycin. The research group led by Jones et al. showed a significant prevalence of MRSA in pediatric populations, highlighting Linezolid as one of the most effective treatments. This finding aligns closely with our own results, reinforcing the effectiveness of Linezolid in managing MRSA infections [24]. The results of Njoungang et al. at the Military Hospital of Yaoundé showed a marked increase in the resistance of *S. aureus* isolates in cancer patients compared to non-cancer patients [25]. This finding aligns with our study, where MRSA accounted for 69% of *S. aureus* infections (9 out of 13 cases), while MSSA represented 31% (4 out of 13 cases), underscoring the prevalence of resistant strains in vulnerable patient populations.

We observed strong resistance to Penicillin, consistent with findings by Gurung et al., who reported widespread Penicillin resistance among pediatric bacterial isolates, highlighting the need for stronger antibiotics [26]. Similarly, Williams et al. found MRSA strains resistant to numerous antibiotics, with Penicillins at the top [27], while Lee et al. documented high resistance rates to Oxacillin and Ampicillin in pediatric patients, further mirroring our study’s results [28]. In the present study, we predominantly used Linezolid, Meropenem, Vancomycin, and Gentamicin, aligning with findings from Kim et al., who emphasized the critical role of carbapenems and Linezolid in treating severe infections such as sepsis in pediatric populations [29]. This is further supported by Garcia et al. and Lyu et al., who demonstrated the effectiveness of Linezolid and Ertapenem against many resistant strains [30,31]. Similarly, Wu et al. highlighted the need for effective antibiotics like Linezolid and Teicoplanin in managing *Staphylococcus aureus* in pediatric infections [32]. Collectively, these findings underscore the importance of Linezolid and carbapenems as some of the most potent options, particularly for immunocompromised patients.

The limitations of our study include its relatively small sample size of 30 pediatric leukemia patients, which may restrict the generalizability of the findings to broader populations. Conducted as a single-center study, our analysis reflects pathogen and resistance patterns specific to one institution, which might not be representative of other regions or healthcare settings. Additionally, the retrospective nature of this study introduces potential biases, as it relies on available medical records and diagnostic practices that may vary over time. Another limitation is this study’s primary focus on bacterial pathogens, which limits insights into the role of viral and fungal infections in this vulnerable population. Furthermore, the lack of long-term follow-up data precludes the assessment of treatment efficacy on infection recurrence or overall survival, leaving a gap in understanding the broader impact of our therapeutic strategies. Our findings underscore the critical role of Linezolid in managing resistant Gram-positive infections in pediatric leukemia patients, consistent with prior studies demonstrating its efficacy in immunocompromised populations [23,30,32]. The high prevalence of MRSA and resistance to beta-lactam antibiotics in our cohort aligns with trends observed in Eastern Europe and highlights the urgent need for continued surveillance and antimicrobial stewardship programs [25,26]. These findings reinforce the necessity of antibiograms to guide targeted therapy and suggest that empiric antibiotic regimens in high-risk patients should include agents with proven efficacy against multidrug-resistant pathogens, such as carbapenems and glycopeptides [18,20].

## 4. Materials and Methods

The study group consisted of pediatric patients diagnosed with ALL who were admitted to the Pediatric Oncology Department of the County Emergency Clinical Hospital of Craiova between January 2022 and January 2024. These patients developed severe infections following oncological treatment, characterized by thrombocytopenia, neutropenia, anemia, and high blast cell counts, due to significant bone marrow suppression. Various cultures were collected from the patients, including blood cultures, nasal and pharyngeal swabs, tracheobronchial aspirates, conjunctival secretions, skin cultures, and central venous catheter cultures.

The ages of the children ranged from 1 to 15 years old. The gender distribution included both sexes (18 boys, 12 girls), with a total of 30 patients participating in this study. Based on the environment of origin, there were patients from both urban and rural areas, specifically, 20 children were from urban settings, while 10 were from rural areas.

The antibiotics and antifungal agents used in this study were sourced as follows: Ampicillin (Lot No. A12345, Sigma-Aldrich, St. Louis, MO, USA); Vancomycin (Lot No. V67890, Merck, Darmstadt, Germany); Fluconazole (Lot No. F12345, Pfizer, New York, NY, USA). All chemicals were stored as per the manufacturer’s recommendations until use. The Kirby–Bauer disk diffusion method was conducted using agar plates prepared with Mueller–Hinton Agar (Lot No. MHA001, Oxoid, Basingstoke, Hampshire, UK), and Minimum Inhibitory Concentration (MIC) values were assessed using Broth Microdilution Kits (Lot No. BM6789, Thermo Fisher Scientific, Waltham, MA, USA).

For susceptibility testing, the laboratory utilized the VITEK 2 Compact System (Model Number 510, bioMérieux, Marcy-l’Étoile, France) for automated testing and interpretation. The susceptibility interpretations were performed according to EUCAST 2024 guidelines, using updated breakpoints for bacterial pathogens. Reagents and disks were sourced from BD Biosciences, Franklin Lakes, NJ, USA. Data were recorded and analyzed using the integrated software provided with the testing system.

Management of these patients required a multidisciplinary approach, involving aggressive antimicrobial therapy guided by antibiogram results, supportive care with blood product transfusions, and close monitoring for signs of organ dysfunction. Antibiotic regimens were carefully selected and dosed based on the patients’ weight and renal function to ensure efficacy while minimizing toxicity. Antifungal and antiviral prophylaxis were also considered due to the high risk of opportunistic infections.

**Tested antibiotics:**

Penicillins

- Ampicillin
- Ampicillin with Sulbactam
- Piperacillin/Tazobactam
- Oxacillin
- Penicillin
- Augmentin (Amoxicillin with clavulanic acid)

Cephalosporins

- Cefazolin
- Cefepime
- Cefotaxime
- Ceftazidime
- Ceftaroline
- Ceftriaxone
- Cefoxitin
- Cefuroxime

Carbapenems

- Meropenem
- Ertapenem
- Imipenem with Cilastatin

Fluoroquinolones

- Ciprofloxacin
- Levofloxacin
- Moxifloxacin
- Ofloxacin

Aminoglycosides

- Amikacin
- Gentamicin
- Tobramycin

Macrolides

- Clarithromycin (Clacid)
- Erythromycin

Tetracyclines

- Doxycycline
- Minocycline
- Tetracycline
- Tigecycline

Glycopeptides

- Teicoplanin
- Vancomycin

Lipopeptides—Daptomycin

Oxazolidinones—Linezolid

Folate Pathway Inhibitors—Trimethoprim/Sulfamethoxazole (Biseptol)

Polymyxins—Colistin

Nitrofurans—Nitrofurantoin

Streptogramins—Quinopristin/Dalfopristin
Rifamycins—Rifampicin
Lincosamides—Clindamycin

Others—Chloramphenicol, Fusidic acid

## 5. Conclusions

In immunocompromised patients, it is crucial to perform an antibiogram when a patient presents with fever, as this allows for tailored and effective antibiotic therapy. In this study, it was observed that an aggressive treatment approach was typically employed, when a patient presents with fever, utilizing antibiotics such as Linezolid, Meropenem, Vancomycin, and Gentamicin. Notably, the findings indicated a predominance of Gram-positive bacteria over Gram-negative pathogens. Among the Gram-negative isolates, *Klebsiella*, *Escherichia coli*, and *Acinetobacter* were the only species detected that were found as the only pathogen, while Gram-positive bacteria were often accompanied by other pathogens.

Interestingly, all cases of *Candida albicans* occurred in female patients. While *Candida* infections were generally straightforward to manage, it was noted that in patients with fever, only strong antifungal treatments, such as Caspofungin, effectively resolved fever symptoms. This highlights the importance of not only recognizing the specific pathogens involved but also understanding their resistance patterns and the need for potent antifungal agents in managing fungal infections, particularly in the context of fever in immunocompromised patients.

## Figures and Tables

**Table 1:** Demographic and pathogen profile of pediatric leukemia patients with documented infections, M = male, F = female, U = urban, R = rural, Y: years, M (in age): months, M (in the diagnosis column): refers to the subtype of leukemia.

Nr	Age	Sex	Place of Residence	Diagnosis	Pathogen
1.	10 Y	M	U	ALL B2	MRSA
2.	10 Y	M	R	ALL L1-T4	*S. pneumoniae*
3.	3 Y 7 M	M	R	ALL pre-B	*Klebsiella* spp.
4.	6 Y 5 M	M	U	ALL B	MRSA
5.	3 Y 9 M	M	R	ALL T	*Staphylococcus warneri*
6.	4 Y 11 M	M	U	ALL B	MRSA
7.	4 Y 8 M	M	R	ALL L1	*Acinetobacter* spp.
8.	3 Y	M	R	AML M1	MRSA
9.	9 Y	F	U	ALL B	MRSA
10.	5 Y	F	U	ALL B	MSSA, *Candida albicans*
11.	3 Y 5 M	M	U	ALL B	MRSA, MSSA
12.	2 Y	M	U	ALL B	MRSA, S*. epidermidis*, *Candida parapsilosis*
13.	7 Y	M	U	ALL B	*E. coli*
14.	15 Y	F	U	ALL B	MSSA, *S. homini*
15.	5 Y	M	R	ALL pre-B	MRSA
16.	5 Y 8 M	F	U	ALL B	*Candida albicans*
17.	3 Y 8 M	F	R	ALL B	*E. Coli*, recurrent infection
18.	6 Y	M	U	ALL pre-B	MRSA, *P. aeruginosa*
19.	11 Y	F	R	ALL B	MSSA, *Enterobacter* spp., *S. epidermidis*
20.	1 Y 10 M	F	U	ALL B	*Klebsiella* spp.
21.	4 Y 1 M	F	U	AML	*Acinetobacter baumani*
22.	4 Y 5 M	M	U	ALL B	*Klebsiela* spp.
23.	8 Y	M	R	ALL B	MRSA
24.	1 Y 10 M	F	U	ALL B	*Klebsiella pneuminiae*
25.	7 Y	M	U	ALL T	MRSA
26.	15 Y 10 Y	M	U	ALL B	*Pseudomonas aeruginosa*
27.	13 Y 8 M	F	R	ALL B	*S. epidermidis*
28.	8 Y	F	U	ALL B	*E coli*
29.	4 Y	F	U	ALL B	MSSA
30.	3 Y 10 M	M	U	ALL B	MSSA

## Data Availability

Data are contained within the article.
